# Transcriptomic analysis of different tissue layers in antler growth Center in Sika Deer (*Cervus nippon*)

**DOI:** 10.1186/s12864-019-5560-1

**Published:** 2019-03-05

**Authors:** Hengxing Ba, Datao Wang, Tung On Yau, Yudong Shang, Chunyi Li

**Affiliations:** 1 State Key Laboratory for Molecular Biology of Special Wild Economic Animals, Institute of Special Wild Economic Animals and Plants, Chinese Academy of Agricultural Sciences, Changchun, 130112 China; 2 Changchun Sci-Tech University, Changchun, 130600 China; 3 College of Science and Technology, Nottingham Trent University, Clifton Campus, Nottingham, NG11 8NS UK

**Keywords:** Antler, Antler growth center, Tissue layer, Chondrogenesis, Transcriptome, RNA-seq

## Abstract

**Background:**

With the unprecedented rapid growth rate (up to 2.75 cm/day), velvet antler is an invaluable model for the identification of potent growth factors and signaling networks for extremely fast growing tissues, mainly cartilage. Antler growth center (AGC) locates in its tip and consists of five tissue layers: reserve mesenchyme (RM), precartilage (PC), transition zone (TZ), cartilage (CA) and mineralized cartilage (MC). The aim of this study was to investigate the transcription dynamics in the AGC using RNA-seq technology.

**Results:**

Five tissue layers in the AGC were collected from three 3-year-old male sika deer using our previously reported sampling method (morphologically distinguishable). After sequencing (15 samples; triplicates/tissue layer), we assembled a reference transcriptome de novo and used RNA-seq to measure gene expression profiles across these five layers. Nine differentially expressed genes (DEGs) were selected from our data and subsequently verified using qRT-PCR. The results showed a high consistency with the RNA-seq results (R^2^ = 0.80). Nine modules were constructed based on co-expression network analysis, and these modules contained 370 hub genes. These genes were found to be mainly involved in mesenchymal progenitor cell proliferation, chondrogenesis, osteogenesis and angiogenesis. Combination of our own results with the previously published reports, we found that Wnt signaling likely plays a key role not only in stimulating the antler stem cells or their immediate progeny, but also in promoting chondrogenesis and osteogenesis during antler development.

**Conclusion:**

We have successfully assembled a reference transcriptome, generated gene expression profiling across the five tissue layers in the AGC, and identified nine co-expressed modules that contain 370 hub genes and genes predorminantly expressed in and highly relevant to each tissue layer. We believe our findings have laid the foundation for the identification of novel genes for rapid proliferation and chondrogenic differentiation of antler cells.

**Electronic supplementary material:**

The online version of this article (10.1186/s12864-019-5560-1) contains supplementary material, which is available to authorized users.

## Background

**Fig. 1:**
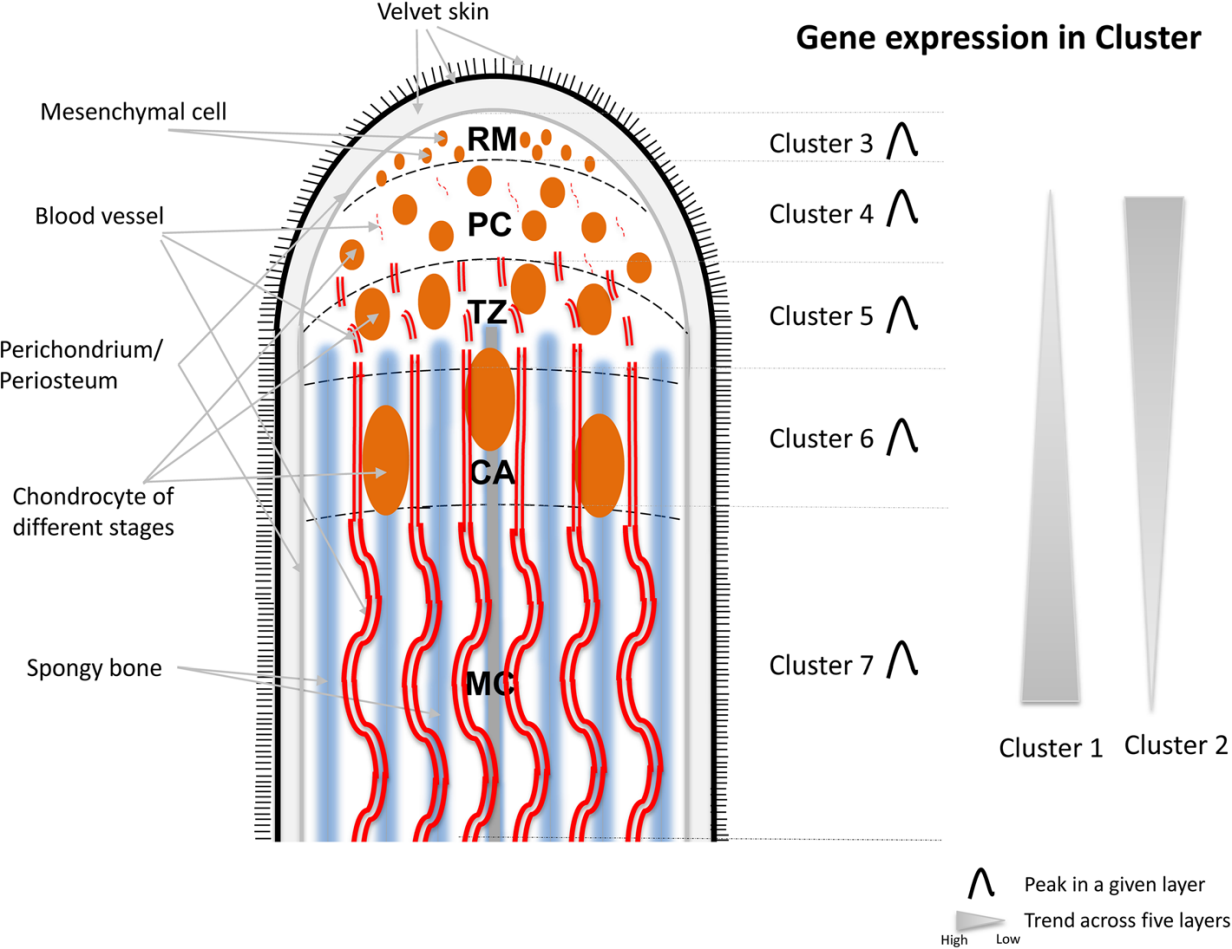
Schematic drawing to show the five tissue layers in an antler growth center. RM: reserve mesenchyme; PC: pre-cartilage; TZ: transition zone; CA: cartilage; MC: mineralized cartilage. This figure also presented in the Fuzzy c-means Clustering Analysis of gene expression patterns in seven clusters (see Results)

A growth system, where normal cells exhibit a rapid proliferation and differentiation without becoming cancerous, would be desirable in identification of potent growth factors, unique signal transduction pathways and novel regulation systems. In this regard, deer antler is an invaluable model to meet these requirements. Antlers are male secondary sexual characteristics and each year form anew from the permanent frontal bony protuberances, called pedicles [1, 2]. During the growth phase, elongation of antlers in some large deer species (such as north America wapiti) can exceed 2 cm/day [3], nonetheless with a well-organized tissue structure [4]. The antler growth center (AGC) is located in the antler tip [5] and histologically consists of five tissue layers from distal to proximal: reserve mesenchyme (RM), pre-cartilage (PC), transition zone (TZ), cartilage (CA) and mineralized cartilage (MC) (Fig. 1) [6, 7].

Antler is an organ of cartilage/bone, and its elongation is achieved through appositional growth [1, 5]. Results from histology, histochemistry, ultrastructure, and BrdU labeling [6, 8, 9] show that the cells in the RM layer are either in mitotically quiescent state (outer sublayer) or at mitotic state (inner sublayer). Below the RM layer, the mesenchymal cells start to differentiate towards a cartilaginous lineage (PC layer), where pre-chondroblasts and newly-formed-isolated vascular streaks reside. Further below the PC layer, pre-chondroblasts gradually maturate toward chondroblasts and chondrocytes; at the same time, the rod-like vascular streaks progressively extend from the two ends in parallel with the long axis of the antler, to form the TZ layer. Within the TZ layer, different stages of cartilaginous cells including prechondroblasts, chondroblasts and chondrocytes co-exist; with different stages of the vascular system (broken and continuous vascular channels co-exist) formation. Finally, continuous cartilage columns (which mainly contain chondroblasts in the periphery and chondrocytes in the center) alternate with continuous vascular channels (functional vascular system) to form the CA layer, the vascularized cartilage (unique feature of antler cartilage). At the end of this proximal layer, chondroclasia, osteogenesis and osteoclasia take place simultaneously to form the MC layer, within which the smooth-surfaced the osteo-cartilage columns are converted into irregular and broken trabeculae [4].

In order to facilitate the discovery of novel genes and/or regulatory systems for rapid antler growth and chondrogenesis using molecular techniques, we established a standardized method to allow rapid and precise sampling of each of these five tissue layers in the AGC of a fresh-cut antler based on morphologically distinguishable markers, and without having to let them undergo histological processes in order to do so [7, 9]. Unfortunately, thus far not much progress has been made in the discovery of the novel genes from the AGC since publication of the method more than a decade ago. This undesirable situation would have been at least partially attributed to the reason that the relevant studies neither used the high-throughput RNA sequencing (RNA-seq) technique, hence a large number of the genes were not detected due to heterologous microarray (deer cDNA versus mouse template) [10, 11]; nor applied the tissue layer sampling method in the study, hence failed to put those identified genes in the biological context [12, 13].

The aim of present study was to use the novel antler model again, but this time to combine the RNA-seq technique with the published standardized tissue layer sampling method, to seek to identify novel genes and regulatory pathways that underpin the system where the fastest growth and chondrogenesis are executed.

## Results and discussion

### Sequencing, de novo assembly and transcript abundance

A total of 654 million (86.58 Gbp) of clean paired-end reads from 15 libraries (triplicates/tissue layer) passed the quality filters (Additional file 1: Table S1). Then, a de novo assembly pipeline was applied to these reads to generate reference transcriptome, because of lacking of a proper sika deer genome at present time (Additional file 2: Figure S1). In total 88,369 non-redundant transcripts (≥ 300 bp) with FPKM ≥0.5 were generated through this process. Based on three different coding sequence prediction methods, a total of 44,177 high-quality coding transcripts were subsequently obtained (Additional file 2: Figure S1, Additional file 3: Table S2). Of these 44,177 high-quality transcripts, the length of N50 (defined as the sequence length of the shortest contig at 50% of the total genome length) was 2533 bp, which is longer than the previously published transcriptomes of the deer [12, 14, 15]. Length distribution results of the assembled transcripts showed that the coding transcripts were more abundant than the non-coding sequences in the bins of long transcripts (Additional file 4: Figure S2A). Transcript abundance was ranged from 3 to 4 orders of magnitude (Additional file 4: Figure S2B), and the coding transcripts (40–50%) were more abundant in the upper ranks of the distribution (Q4) than the non-coding sequences (20–30%) (Additional file 4: Figure S2C). Core Eukaryotic Genes Mapping Approach (CEGMA) [16] was applied to evaluate completeness of our transcript assembly, and found that a high percentage of core genes (94.35% had complete sequences; only 5.25% were partially sequenced) presented in our transcriptome data.

As a gene can have multiple transcript isoforms with different length, the longest transcript of a gene in this study was selected to represent that gene. A total of 13,203 genes were generated and then annotated using the top 1 orthologous of all currently known species based on UniProt database (May 2017), including human, mouse, cattle and sheep. We further screened the characteristics of the assembled 13,203 gene sequences by comparing them with protein sequences (not incorporated in UniProt) derived from genomes of closely related species, i.e., red deer [17], white tailed deer and cattle. For this analysis, BLASTX v2.5.0+ with default search parameters was carried out. The results showed that 13,131 (99.5%) and 13,110 (99.3%) out of 13,203 genes passed BLAST matched thresholds of E-value ≤10^− 5^ for cattle and white tailed deer respectively. But, the matched ratio with red deer proteins (12,145, 92.0%) was relatively low (Additional file 5: Table S3). The possible reason for this is that the current red deer genome is still not a completed version (1.96 Gbp in total). Our results also suggested that ~ 0.5–0.7% gene sequences could present the low conservativeness between sika deer and these closely related species. Interestingly, the slight redundancy sequences (~ 3.2%, Additional file 5: Table S3) could reside in these 13,203 genes although our transcriptome dataset was clustered using cd-hit-est tool (see methods). In addition, our results showed that ~ 60% out of deer genes is likely to be expressed in the AGC (Additional file 5: Table S3), which is compatible with the number of genes expressed in other mammalian tissues (Fig. 1 refer to [18]). Overall, a high-quality reference gene dataset (13,203 genes) was successfully generated, and was further used for the downstream bioinformatics analysis in this study (Additional file 6: Figure S3).

### Gene expression profiling matches developmental state of tissue layers

**Fig. 2:**
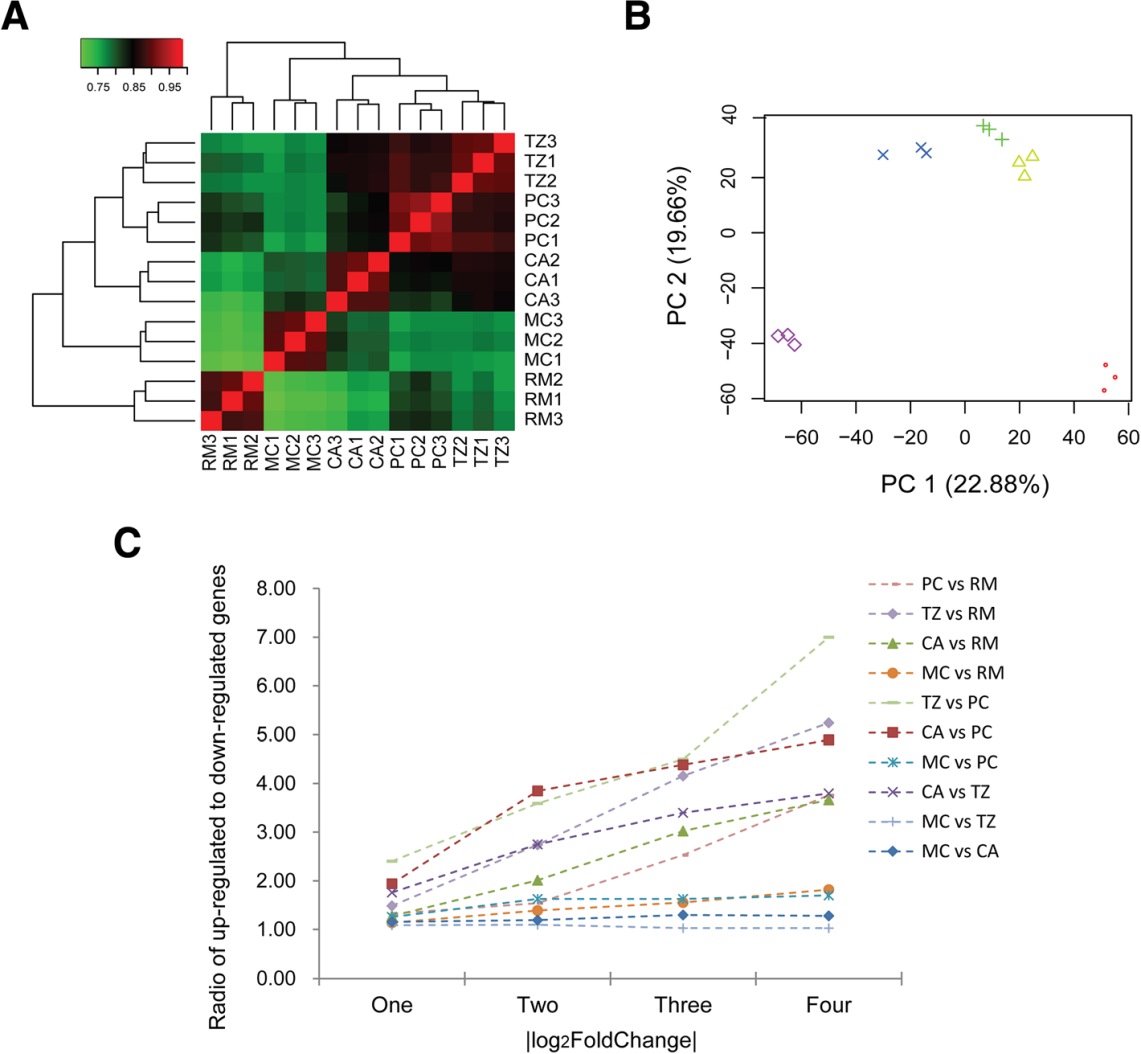
Comparison of gene expression across five tissue layers in the antler growth center. **a**) Pairwise Pearson Correlations of gene expression across 15 samples (triplicates/each tissue layer). **b**) Principal Component Analysis of gene expression across 15 samples. **c**) Ratio of up-regulated to down-regulated genes across five tissue layers on different thresholds (|log2FoldChange|≧1, 2, 3 and 4)

To assess the global variation in gene expression across the AGC tissue layers, a matrix to the normalized expression values (mapping reads, 13,203 genes) were conducted. The results of pairwise Pearson Correlation indicated that the RM layer showed a stand lone group; whereas the rest of other four layers (PC, TZ, CA and MC) clustered together as a composite group, although the first three layers (PC, TZ and CA) in the composite group were closer compared to the last layer (MC) (Fig. 2A). This finding may reflect the use of distinct regulatory genes in the RM layer compared to the other layers, possibly because the cells in the RM layer have stem cell attributes while cells in the other layers are in the different differentiating states. Principal Component Analysis were also performed, and the results showed that PC1 explained 22.9% of the overall variation, which was found too low to separate the five tissue layers but could separate the RM layer (outmost) from the MC layer (innermost). PC2 explained 19.7% of the overall variation, which was found to allow either the RM layer or the MC layer to be distinguishable from the composite group (PC, TZ and CA) (Fig. 2B). Interestingly, the PC1 variation revealed a gradient of layer samples that perfectly fits their position in a proximo-distal axis of the AGC. Overall, our results suggest that the variation trajectory follows the similar pattern of the tissue layers in the AGC.

In order to detect the changes in ratio of up-regulated to down-regulated genes across the five tissue layers inversely (i.e. a proximal layer vs its every distal layer), we used the four pre-set levels of thresholds (|log2FoldChange|≧1, 2, 3 and 4). The results showed that the higher the level the bigger the ratio (Fig. 2C), suggesting that the elevated level of up-regulated genes is positively correlated with the degree of cell differentiation (mesenchymal cells to chondrocytes) in the AGC.

### Extensive changes detected in functional categories across tissue layers

**Fig. 3:**
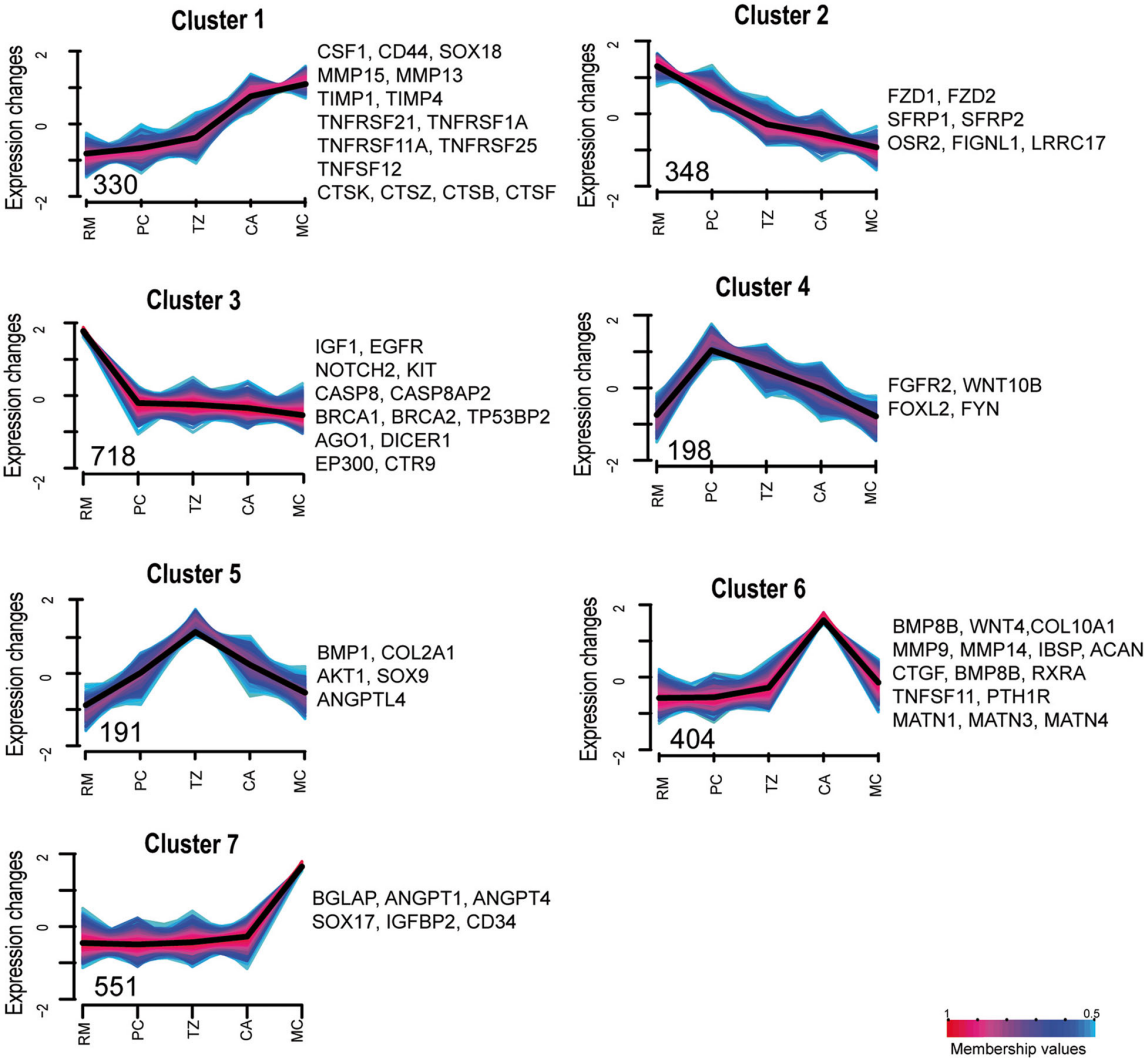
Fuzzy c-means clustering identifies general patterns of gene expression across the five tissue layers in the antler growth center (also refer to Fig. 1). The average FPKM values of DEGs (|log_2_FoldChange| ≥ 1.5, adjusted Pvalue ≤0.001) are used. The MS of a given gene within a cluster is represented in color, with red (MS = 1) indicating high association. The number of genes with MS ≥ 0.5 in clusters is also shown. For each cluster, the key genes involved in significantly enriched GO BP categories (Additional file 7: Table S4) were shown

To investigate the large-scale patterns of gene expression across the five tissue layers, Fuzzy c-means clustering model was used to group 5585 DEGs (selected based on this criteria (|log_2_foldchange| ≥ 1.5, adjusted Pvalue ≤0.001)), and these DEGs were grouped into seven clusters. Of these 5585 DEGs, 2740 were selected based on their member score (MS) within the seven clusters (MS ≧ 0.5) and used for further analysis (Fig. 3, also refer to Fig. 1). Next, we performed GO enrichment analyses using genes in each cluster to identify key biological process (BP) categories (Additional file 7: Table S4). The genes in Cluster 1 mainly included those with expression levels steadily increasing over sequential differentiation processes in the AGC (from the RM layer to MC layer). As expected, these genes were mainly related to key BP categories, e.g., extracellular matrix disassembly (EASE score = 0.002, e.g., CTSK, CD44, MMP13 and MMP15), osteoclast differentiation (EASE score = 0.007, e.g., CSF1 and TNFRSF11A), angiogenesis (EASE score = 0.015, e.g., TNFSF12 and SOX18) and wound healing (EASE score = 0.049, e.g., TIMP1). The genes in Cluster 2 included those showing an opposite trend in expression level to Cluster 1; these genes were mainly related to Wnt signaling and cell polarity (EASE score = 0.033, e.g., FZD1, FZD2, PSMA1 and PSMA4), and osteoblast proliferation (EASE score = 0.002, e.g., OSR2). These findings are consistent with our histological observations in that the transition from undifferentiated mesenchymal cells to chondrocytes distoproximally across the tissue layers is gradual, along with vascularization, chondroblast maturation and chondrification [9]. In addition, these genes may also play a role in the formation of vascularized cartilage, a unique structure which is thought to be required to meet the metabolic demands for rapidly growing antler tissue [6, 9] and as a conduit for hemopoietically derived chondroclast and osteogenic progenitors [19].

For clusters 3, 4, 5, 6 and 7, up-regulated layer-specific genes were found to be with expression peaks in each of the five tissue layers (RM, PC, TZ, CA and MC layers). The genes in Cluster 3 had expression peaks in the RM layer and were found to be involved in cell proliferation (EASE score = 0.009, e.g., IGF1, EGFR), stem cell population maintenance (EASE score = 0.044, e.g., NOTCH2 and KIT), regulation of apoptotic process (EASE score = 0.02, e.g., CASP8, CASP8AP2 and TP53BP2) and DNA repair (EASE score = 0). The rate of antler growth can reach 2 cm per day [3], and this rapid growth is achieved mainly through fast cell proliferation in the RM layer [6, 9]. The rapid rate of cell proliferation in antlers would not only require factors that stimulate fast cell proliferation, but also those that properly control the cell cycle and protect genomic stability. It is also notable that genes that were involved in epigenetic regulation, including gene silencing by RNA (EASE score = 0.001, e.g., AGO1 and DICER1) and histone modification (EASE score = 0.01, e.g., CTR9 and EP300) were found in the RM layer, strongly implying early imprinting of the stem cell populations in this layer.

The genes in Cluster 4 showed an expression peak in the PC layer, and were found to be related to positive regulation of Wnt signaling (EASE score = 0, e.g., FGFR2 and WNT10B). The PC layer mainly consists of newly differentiated prechondroblasts, which actively form and secrete extracellular matrix [8]. Wnt signaling was detected in this Cluster (also in Clusters 2 and 3), suggesting this signaling pathway could be involved in early antler cell differentiation toward chondrogenesis.

The genes in Cluster 5 showed an expression peak in the TZ layer, and were found to be related to cartilage condensation (EASE score = 0.02, e.g., BMP1, SOX9 and COL2A1) and response to hypoxia (EASE score = 0.03, e.g., AKT1 and ANGPTL4). In the TZ layer, cells are in the differentiation transition from prechondroblasts to chondroblasts and chondrocytes, with vascular channels progressing from broken (unfunctional) to continuous (functional) [9]. Cartilaginous cells at different stages of differentiation in the vicinity of unfunctional vascular channels (not yet linked to the body vascular system), or further away from the functional vascular system (linked to the body vascular system) would be subjected to low oxygen tension/hypoxia. It is not surprising that, in order to cope with this low oxygen tension, these cells would have to highly express genes that are responsive to hypoxia.

The genes in Clusters 6 showed expression peaks in the CA layers. As expected, these genes were found to be related to extracellular matrix organization (EASE score = 0, e.g., COL10A1 and ACAN), angiogenesis (EASE score = 0.004, e.g., MMP14), regulation of chondrocyte differentiation (EASE score = 0.015, e.g., CTGF), ossification (EASE score = 0.03, e.g., MMP9 and TNFSF11) and bone mineralization (EASE score = 0.006, e.g., PTH1R), and response to hormones (EASE score = 0.03, e.g. LYN). The genes in Clusters 7 had expression peaks in the MC layers, and were found to regulate osteoclast differentiation (EASE score = 0.044, e.g., BGLAP) and negative regulation of blood coagulation (EASE score = 0.044, e.g., CD34). These findings support the previous ultrastructural observations, in which almost all hypertrophic chondrocytes were eventually subject to degeneration and apoptosis [8]. Programmed cell death of hypertrophic chondrocytes in both the CA and MC layers would create more space for the brought-in-osteogenic progenitor cells to build up bone tissue [20]. The seasonal high levels of androgen hormones would initiate the final antler mineralization processes, further result in the interruption of the blood flow into antlers from their bases, and cause eventual demise of antlers [21, 22]. Overall, gene expression profiling across the five tissue layers within an AGC matched the developmental states in each corresponding tissue layer.

### Hub genes detected using co-expression network analysis and associated with Chondrogenesis and angiogenesis

**Fig. 4:**
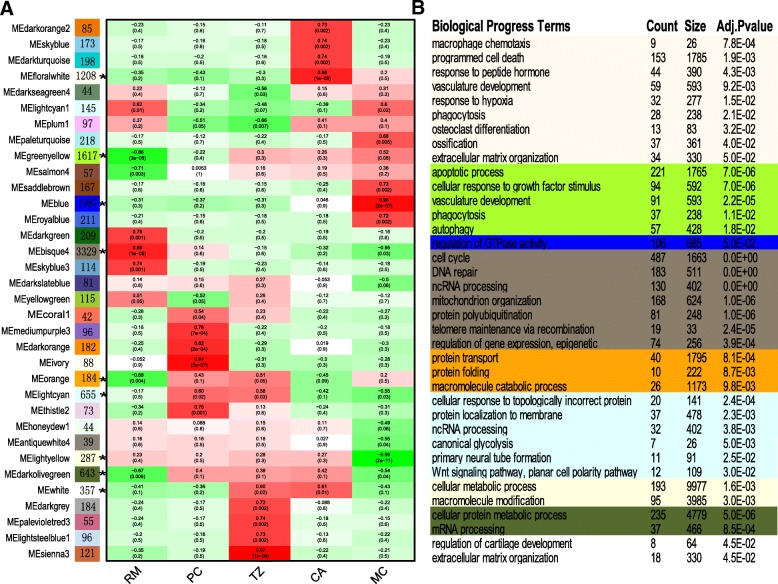
Co-expression gene networks. **a**) Genes (13,103) are assigned to 34 different modules (39 to 3329 genes/module). Nine modules marked with asterisks have significant BP categories identified by a hypergeometric test after adjusted Pvalue ≤0.05 and show significant negative/positive module-trait correlations with the corresponding tissue layers (|Pearson Correlation| ≥ 0.6 and Pvalue ≤0.01). **b**) For these nine modules, significantly enriched GO biological process categories are shown (Additional file 8 Table S5). For clarity, only the selected key categories are shown in the figure

Sequences of the obtained 13,203 genes were also analyzed to investigate the association with the chondrogenesis and angiogenesis in the AGC using Weighted Gene Co-expression Network Analysis (WGCNA). The results showed that majority of these genes (13,103 genes; 99.24%) were assigned to 34 modules (39–3329 genes per module). Of these 34 modules, nine (10,267 genes: 78.36%, marked with asterisk in Fig. 4A) were found to have significantly negative/positive module-trait correlations with the corresponding tissue layers when the |Pearson Correlation| ≥ 0.6 and Pvalue ≤0.01 criterion was applied, and to be enriched in the BP categories (Fig. 4B, Additional file 8: Table S5). These BP categories were concordant with the counterparts generated from our Fuzzy c-means clustering analysis. For example, the MEfloralwhite module showed a positive module-trait correlation with the CA layer and these enriched BP categories were consistent with those in Cluster 6, and partially in Cluster 1. Likewise, the MElightcyan module showed a positive module-trait correlation with the PC layer, such as Wnt signaling pathway, which is consistent with that in Cluster 4.

**Fig. 5:**
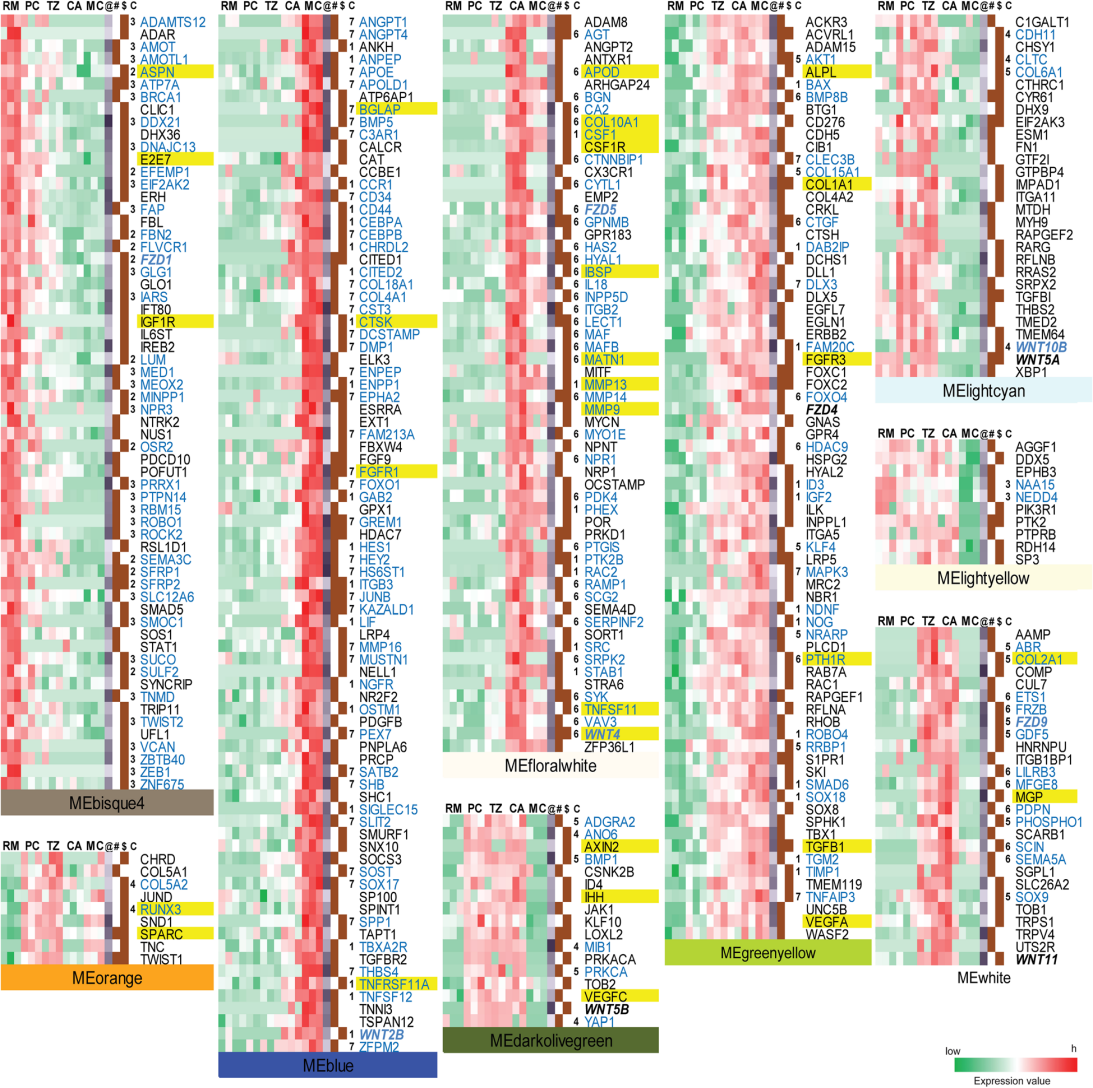
Expression of 370 highly connected intra-module hub genes across the five tissue layers. All hub genes are derived from nine modules marked with asterisks in Additional file 6: Figure S3A with high MM values (MM ≥ 0.7). Each line represents the scaled log_2_ transition of average FPKM value for each individual gene. Among them, 29 genes being highlighted with yellow are reported previously and 187 genes that emphasized with blue font are involved in seven Fuzzy c-means clusters. Particularly, the Wnt related genes and their receptors (Frizzled genes) are emphasized with bold italic. ‘@’: MM value ranged from 0.7 to 1. ‘#’: genes related to angiogenesis. ‘$’: genes related to cartilage/bone development. ‘C’: Cluster ID related to Fuzzy c-mean Clustering Analysis

Next, we focused on highly connected intra-module hub genes in each module, and these genes had high module membership (MM) values (MM ≧ 0.7) in their respective modules. We detected 370 hub genes that might be involved in rapid antler growth, and in regulation of chondrogenesis and angiogenesis (Fig. 5). Of these hub genes, 187 (50.5%) were involved in seven Fuzzy c-means clusters, and more specifically, these associated hub genes from each module belong to one or two clusters (except for 26 genes in MEgreenyellow module), further suggesting that the results between WGCNA and Fuzzy c-means analysis are consistent. Twenty nine hub genes are also shown in the previously published studies using other molecular technologies (e.g., western blot, qRT-PCR, in-situ hybridization or cDNA microarrays) (Table 1); and were found to be involved in extracellular matrix organization, mineralization and degradation (e.g., COL1A1, COL2A1, COL10A, MMP9, MMP13, SPARC, IBSP, BGLAP, CTSK, ALPL MGP and MATN1), signaling molecules (e.g., IGF1, VEGF, IHH, WNT4, PTHLH, CSF1, TNFSF11 and TGFB1), receptors (e.g., FGFR1, FGFR3, PTH1R, CSF1R, CALCR and TNFRSF11A), binding (e.g., APOD) and transcriptional factors (e.g. RUNX3 and SOX9). Some of the molecules and their receptors (i.e. FGFR1, FGFR3, IHH, SOX9, PTHLH and PTH1R) in our hub gene pool are found to be expressed during embryogenesis, and reportedly regulate long bone formation and growth plate development [23, 24]. This finding supports a previous hypothesis that there is evolutionary conservation of the developmental signaling pathways that occur during embryogenesis and postnatal cartilage/bone regeneration [25].

**Table 1:** Summary of data for 29 hub genes reported in the previous studies of antler tip using molecular technologies

Gene symbol	Gene name	Biological effect in antler tip growth	Reference
IGF1R	insulin like growth factor 1 receptor	Increases antler mesenchymal cell proliferation	[44, 45, 46]
COL1A1	collagen type I alpha 1 chain	Localizes to a subset of flattened cells on the periphery of the trabeculae	[47, 48]
COL2A1	collagen type 2 alpha 1 chain	Expresses transiently in the antler tip, as a marker of mature chondrocytes	[47]
COL10A1	collagen type X alpha 1 chain	Increases by recently differentiated chondrocytes and continued to be expressed throughout the unmineralized and mineralized cartilage region, as a marker of hypertrophic chondrocytes	[47]
MMP9	matrix metallopeptidase 9	Highly expresses in the cells resident in antler cartilage, involved in matrix degradation	[47, 50]
MMP13	matrix metallopeptidase 13	Highly expresses in the chondrocytes, involved in matrix degradation	[49]
MATN1	matrilin 1, cartilage matrix protein	Increases only in the cartilage regions, as a marker of prehypertrophic and hypertrophic chondrocytes	[51]
VEGFA/C	vascular endothelial growth factor	Expresses from precartilage to cartilage regions, having an angiogenic effect within antler	[52]
IHH	indian hedgehog	Localizes only in recently differentiated chondrocytes, involved in the control of chondrocyte differentiation	[25]
PTHLH/PTH1R	parathyroid hormone 1 and its receptor	Expresses highly in cartilage, promote the proliferation and differentiation of chondrocytes, also localized in cells of the osteoclast lineage	[53][54]
WNT4	Wnt family member 4	Regulates terminal differentiation of antler chondrocytes	[55]
CSF1/CSF1R	colony stimulating factor 1 and its receptor	Induces osteoclastogenesis in vitro	[50]
BGLAP	bone gamma-carboxyglutamate protein	Highly expresses in chondrocytes, as an indicators of bone mineral metabolism	[56]
ALPL	alkaline phosphatase	Inhibitors of mineral deposition, increases indicating differentiation into osteoblast-like cells, as a marker of osteoblast differentiation	[57]
SPARC	secreted protein acidic and cysteine rich	Expresses highly in chondrocytes, as an indicators of bone mineral metabolism	[49, 56]
TNFSF11/ TNFRSF11A	tumor necrosis factor superfamily member 11/TNF receptor superfamily member 11a (RANKL/RANK)	Induces antler osteoclastogenesis in vitro	[50]
FGFR1	fibroblast growth factor receptor 1	Widely expresses in the integument and osteocartilaginous compartments	[58]
FGFR3	fibroblast growth factor receptor 3	Widely expresses in the integument and osteocartilaginous compartments	[58]
CTSK	cathepsin K	Expresses only in osteoclasts, involved in matrix degradation	[50]
CALCR	calcitonin receptor	Expresses only in osteoclasts, the most specific marker of the osteoclast lineage cells	[50]
IBSP	integrin binding sialoprotein	Highly expresses in cartilage regions	[56]
TGFB1	transforming growth factor beta 1	Regulation of chondrogenesis	[25]
MGP	matrix Gla protein	Highly expresses in cartilage regions	[56]
RUNX3	runt related transcription factor 3	Involved in regulating the differentiation of chondrocytes	[59]
APOD	Apolipoprotein D	Expresses primarily in chondrocytes,	[60]
SOX9	SRY-Box 9	Highly expresses in proliferating and prehypertrophic chondrocytes	[61]

Six Wnt genes (WNT2B, WNT5A, WNT5B, WNT4, WNT10B and WNT11), and four their receptor genes (Frizzled genes; FZD1, FZD4, FZD5 and FZD9) in our hub gene pool were overrepresented. It is known that the Wnt signaling pathway plays an essential role in cartilage/bone development during embryogenesis [26–28]. Furthermore, the canonical Wnt signaling pathway is known to be involved in the establishment of AGC in the early antler regeneration, more specificallyβ-catenin is an important factor controlling survival and lineage specification of the mesenchymal progenitor cells toward chondrogenesis [29]. In our results, both Wnt genes and their receptors were identified and fell in seven of nine co-expressed modules, suggesting that this pathway may not only play an important role in promoting the proliferation of the stem cells or their immediately differentiated progeny in the AGC, but also be involved in chondrogenesis during antler development. Therefore, we think that further studies should be directed at elucidation of the mechanisms underlying the cross-interactions between Wnt signaling pathway and other local signaling pathways, such as TGF-beta and Hedgehog signaling pathways, as well as hormonal stimuli, for the regulation of antler cell proliferation, survival and chondrogenesis.

Four Fox genes (FOXC1, FOXC2, FOXO1 and FOXO4) and four Sox genes (SOX8, SOX9, SOX17 and SOX18) were found to be overrepresented in our hub gene pool. FOXC2 is reported to augment tumor propagation and metastasis in osteosarcoma [30]. In contrast, FOXO1 and FOXO4 are regarded as tumor suppressor genes for certain cancers including osteosarcoma through diversified mechanisms, such as initiating apoptosis [31]. Antler growth involves fast cell proliferation that is elegantly regulated without becoming cancerous, suggesting FOXO genes play a role in the maintenance of normal antler tissue growth.

**Fig. 6:**
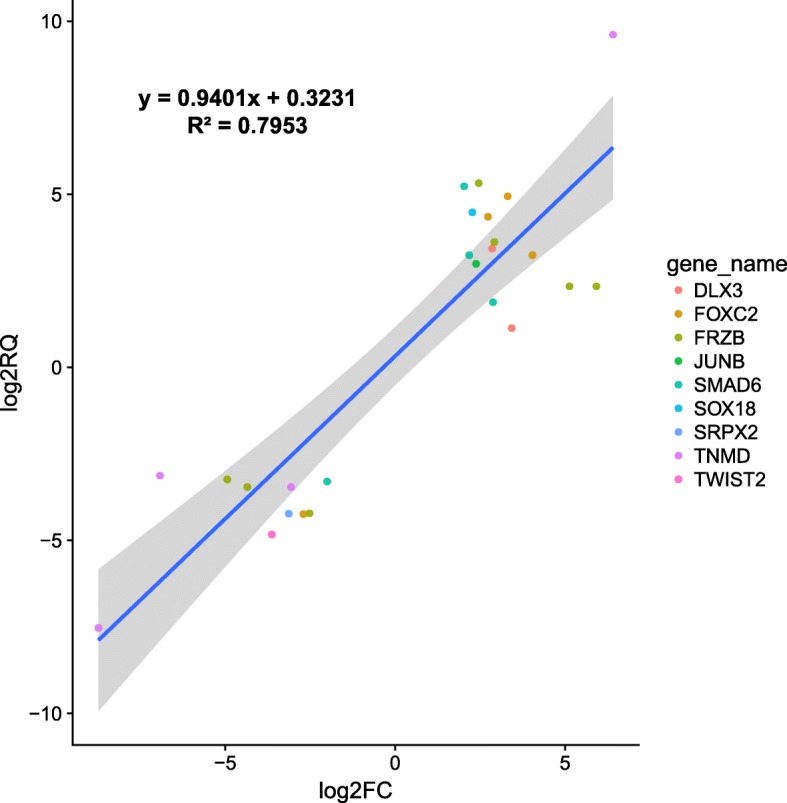
Correlation between RNA-seq and qRT-PCR for nine highly differentially expressed hub genes. Each color dot represents a qRT-PCR pairwise reaction with the corresponding gene

In order to verify our RNA-seq results, nine hub genes (i.e., DLX3, FOXC2, FRZB, JUNB, SMAD6, SOX18, SRPX2, TNMD and TWIST2) were selected from our hub gene pool (370 hub genes in total) based on the criteria of |log2foldchange| ≧ 2 and adjusted Pvalue ≤0.001, and validated using qRT-PCR. Results of 25 pairwise PCR reactions showed high consistency with that of RNA-seq data (R2 = 0.80) (Fig. 6).

## Conclusions

Overall, we believe our results (such as 370 hub genes involved in nine co-expressed modules, particular genes that predominantly expressed in and highly revenant to each tissue layer) provide a foundation for future studies of more detailed molecular analyses to the development of the AGC. Besides, our transcriptome data would be valuable for other functional genomic research in sika deer or closely-related species. In the long term, establishing the molecular and cellular mechanisms involved in regulating chondrogenesis may lead to the development of strategies for enhancing cartilage/bone repair and regeneration in other mammals including humans.

## Methods

### Sample collection

Antler tissues were collected from three 3-year-old healthy sika deer (*Cervus nippon*) at about 30 days after casting of the previous hard antlers, and processed as previously described [9]. Briefly, the distal 8 cm of the growing tip was removed and sectioned sagittally along the longitudinal axis. Five layers of tissues of the tip were immediately dissected and then cut into 4–6 mm pieces, which were then frozen in liquid nitrogen and stored at − 70 °C for RNA preparation and sequencing.

### RNA preparation and sequencing

The tissue samples were rapidly grinded into fine powder by using Freezer/Mill 6770 (SPEX CertiPrep Ltd., USA). Total RNA was extracted from the sample powder using a Trizol reagent (Invitrogen Inc., Camarillo, CA) according to the manufacturer’s procedure. RNA quality was confirmed using Bioanalyzer with a minimum RNA integrity number of 7.0. Six micrograms of total RNA were used to construct libraries according to the manufacturer’s instructions (Illumina TruSeq Library Preparation Kit v3). Libraries were sequenced using an Illumina HiSeq X Ten at BGI (Shenzheng, China). We sequenced three biological replicates of each layer of tissue with 150 bp paired-end sequencing.

### Transcriptome assembly, annotation and differential expression

We used Trinity v2.4.1 [32] with fixed default k-mer size of 25 to carry out de novo assembly of a reference transcriptome from the quality-filtered reads. The paired-end reads were mapped to the assembled transcriptome with Bowtie2 v2.0.5 (−no-mixed --no-discordant --gbar 1000 --end-to-end -k 200) [33], and abundance estimation (FPKM, fragments per kilobase of transcript per million mapped reads) was performed using RSEM v1.3.0 [34]. Next, we developed a strict pipeline to filter the assembly errors and background sequences (Additional file 2: Figure S1). Briefly, 1) Removal of short sequences (length ≤ 300 bp); 2) Removal of the background sequences (FPKM of all replicates in any one tissue ≥0.5); 3) Removal of the redundancy transcripts (identity ≥95%) using cd-hit-est v3.0.3 [35]; 4) Removal of non-coding transcripts. The coding transcripts (≥ 100 amino acid) were predicted by ESTScan v2.2.1 [36] with human model and TransDecoder v2.0.1 [32], and further annotated by searching against UniProt database using BLASTX (E-value ≤10^− 5^). Finally, differential gene expression analysis was conducted based on the mapped counts using DESeq2 v2.1.18 R package [37] at an adjusted Pvalue of 0.001.

### Fuzzy c-means Custer analysis

The mean FPKM values were clustered using Fuzzy c-means clustering from Mfuzz v2.42 R package [38]. Only genes with significant differences in expression between at least two layers (|log_2_FoldChange| ≥ 1.5, adjusted *p*-value ≤0.001) were used as input for this clustering analysis. The optimal number of clusters was set to 7 and the fuzzifier coefficient set to 2.01. The number of clusters was determined at which the minimum centroid distance plateau was achieved using the Dmin function (Additional file 9: Figure S4). Genes with a membership score (MS) of at least 0.5 were plotted and used as input for categorical enrichment analysis.

### Weighted gene co-expression network analysis

A co-expression network was constructed using WGCNA v1.48 R package [39]. In brief, the gene FPKM matrix was subjected to a variance stabilizing transformation using DESeq2 v2.1.18 R package [37]. A soft-threshold power value of 16 was selected in this analysis, which corresponds to R^2^ (> 0.9). Co-expression modules were identified as clusters from the dendrogram using the cutreeDynamic function with a minimum module size of 30 genes. Modules with eigengene correlations no less than 0.75 were subsequently merged using the mergeCloseModules function with a height cutoff of 0.25. We then conducted module-trait correlations between the module eigengenes and libraries corresponding to each of the developmental layers.

### Gene ontology enrichment analysis

We used two bioinformatics tools (DAVID version 6.8 website [40] and GOstats v2.44 R package [41]) to perform GO enrichment analysis for the gene set from Fuzzy c-means cluster and WGCNA analysis. The DAVID version 6.8 website was used to obtain overrepresented GO BP categories with an adjusted Fisher exact *P* value (EASE score). The GOstats v2.44 R package was performed with Benjamini and Hochberg correction using p.adjust program in R package. The human orthologs of the corresponding deer genes were used in the GO enrichment tests to take advantage of the more complete GO annotation available for human genes.

### Quantitative real-time PCR (qRT-PCR) analyses

To confirm the DEGs identified from RNA-seq assay, nine highly expressed genes (see results) with great alteration expression levels were chosen and validated using qRT-PCR. The specific primers located in gene coding regions were designed using Primer 5 software and listed in Additional file 10: Table S6. Actin, cytoplasmic 1 (ACTB) was used as a standard control according to our in-house selection standard. Total RNA was firstly treated with DNase I before reverse transcription by superscript III double-stranded cDNA synthesis kit (Invitrogen Inc., Camarillo, CA). The qRT-PCR was then performed using the SYBR Kit (Applied Biosystems, Foster City, CA, USA) according to the manufacturer’s protocol using an Applied Biosystems 7500 detection system. The melting curves for verification of amplification specificity by a thermal denaturing step. The relative quantitative method (2^-ΔΔCT^) was used to calculate the fold change in the expression levels of target genes [42]. All reactions were performed in three biological replicates using the independent RNA samples. Linear regression analysis and loess smooth plot were performed by ggplot2 R package [43].

## Additional files

Additional file 1: **Table S1.** Summary of sequencing data of 15 samples across the five tissue layers. (XLSX 11 kb)

Additional file 2: **Figure S1.** The flow chart illustrates the major steps involved in the de novo reference transcriptome assembly to filter out the low quality and error assembled transcripts. (JPG 5583 kb)

Additional file 3: **Table S2.** Statistics of transcript assembly in the pipeline. (DOCX 16 kb)

Additional file 4: **Figure S2.** De novo assembly of the reference transcriptome. **A)** Length distribution of coding and noncoding transcripts across the length bins. Triangles and dots represent the percentages of coding and noncoding transcripts in the length bins, respectively. **B)** Expression levels displayed differences of 3–4 orders of magnitude. For each tissue layer, transcripts with FPKM ≥0.5 are all replicates. **C)** In all cases, the coding transcripts (40–50%) are more abundant in the upper ranks of the distribution (Q4) than the non-coding sequences (20–30%). (PDF 468 kb)

Additional file 5: **Table S3.** Summary of 13,203 gene sequences matched with non-redundancy proteins of three closely related species using BLASTX. (DOCX 14 kb)

Additional file 6: **Figure S3.** The flow chart shows the steps involved in down-stream bioinformatics analysis, including Principal Component Analysis, Fuzzy c-mean Clustering Analysis and WGCNA. The blue boxes represent executive bioinformatics tools with the corresponding threshold values and the white boxes represent the statistical results from tools. (JPG 5070 kb)

Additional file 7: **Table S4.** GO BP enrichment of the DEGs of seven Fuzzy c-means clusters. (XLSX 72 kb)

Additional file 8: **Table S5.** GO BP enrichment of the genes of nine WGCNA modules. (XLSX 508 kb)

Additional file 9: **Figure S4.** Plot of the minimum centroid distance across a range of cluster number (JPG 3924 kb)

Additional file 10: **Table S6.** Primers of the nine hub genes were used for qRT-PCR. (XLSX 9 kb)

## Abbreviations

AGC: Antler growth center
BP: Biological process
CA: Cartilage
CEGMA: Core Eukaryotic Genes Mapping Approach
DAVID: Database for Annotation, Visualization and Integrated Discovery
DEGs: Differentially expressed genes GO: gene ontology
FPKM: Fragments per kilobase of transcript per million mapped reads
MC: Mineralized cartilage
MM: Module membership
MS: Membership score
PC: Pre-cartilage
qRT-PCR: Quantitative reverse transcription poly chain reaction
RM: Reserve mesenchyme
RNA-Seq: RNA sequencing
TZ: Transition zone
WGCNA: Weighted correlation network analysis
