# Supplementary material for: Transcriptomic analysis of different tissue layers in antler growth Center in Sika Deer (Cervus nippon)
Source: BMC Genomics. 2019 Mar 5;20:173. doi: 10.1186/s12864-019-5560-1 (PMC6402185; doi:10.1186/s12864-019-5560-1)
Supplement: Supplementary file 3 — Table S2. Statistics of transcript assembly in the pipeline. (DOCX 16 kb) [file 12864_2019_5560_MOESM3_ESM.docx]

**Table S2 Statistics of transcript assembly in the pipeline.**

|  | **Total length (bp)** | **Total number**  **(≥300bp)** | **Total number**  **(≥1kp)** | **Total number**  **(≥2kp)** | **Average length** | **N50** |
| --- | --- | --- | --- | --- | --- | --- |
| **Trinity assembly** | 252,923,736 | 168,935 | 74,824 | 42,127 | 1,497 | 2,677 |
| **High quality transcript** | 169,157,473 | 103,974 | 52,306 | 29,165 | 1,626 | 2,730 |
| **Non-redundant transcripts** | 133,302,190 | 88,369 | 41,096 | 21,901 | 1,508 | 2,533 |
| **Coding transcripts** | 103,877,873 | 48,231 | 33,680 | 20,194 | 2,153 | 3,113 |
| **Non-coding transcripts** | 29,464,455 | 40,138 | 7,424 | 1,710 | 734 | 848 |
