# Supplementary figures and images for: Transcriptomic analysis of different tissue layers in antler growth Center in Sika Deer (Cervus nippon)
Source: BMC Genomics. 2019 Mar 5;20:173. doi: 10.1186/s12864-019-5560-1 (PMC6402185; doi:10.1186/s12864-019-5560-1)

A

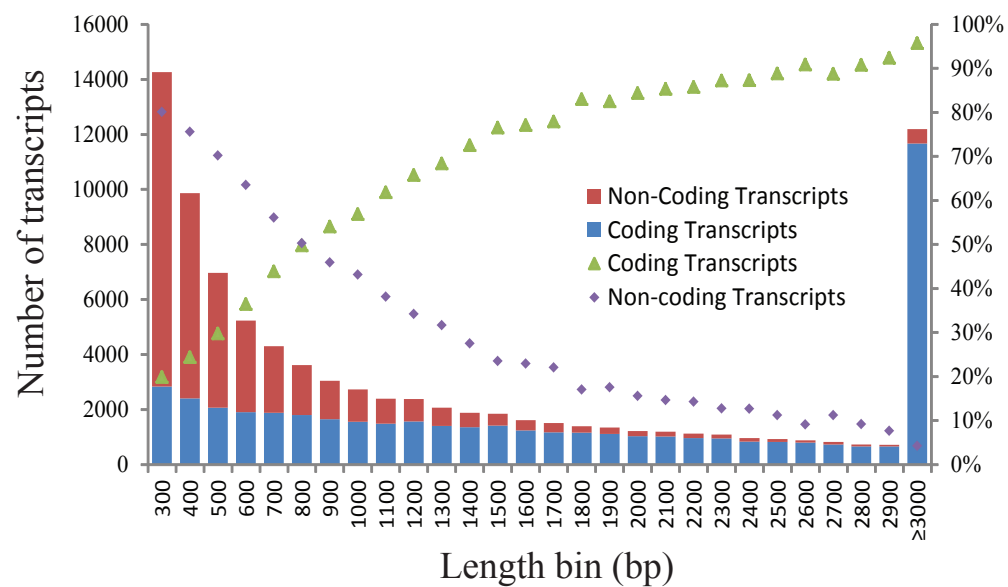

B

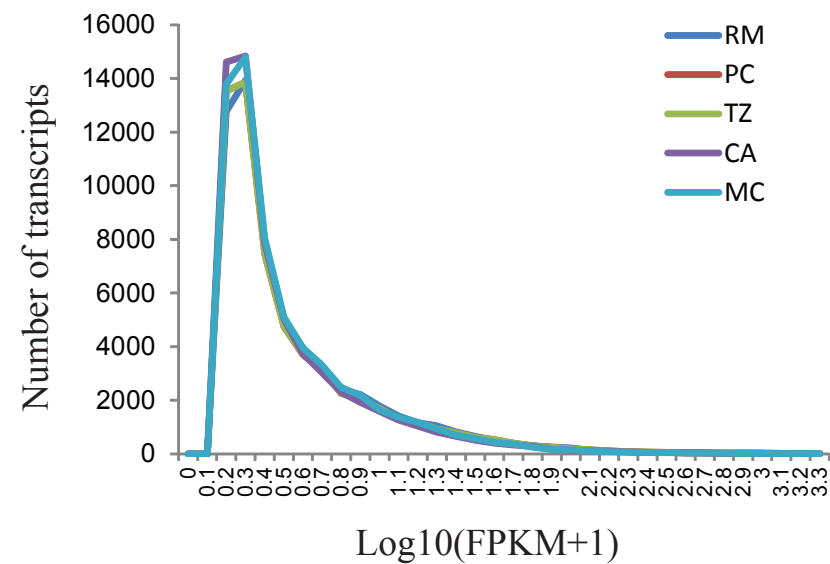

C

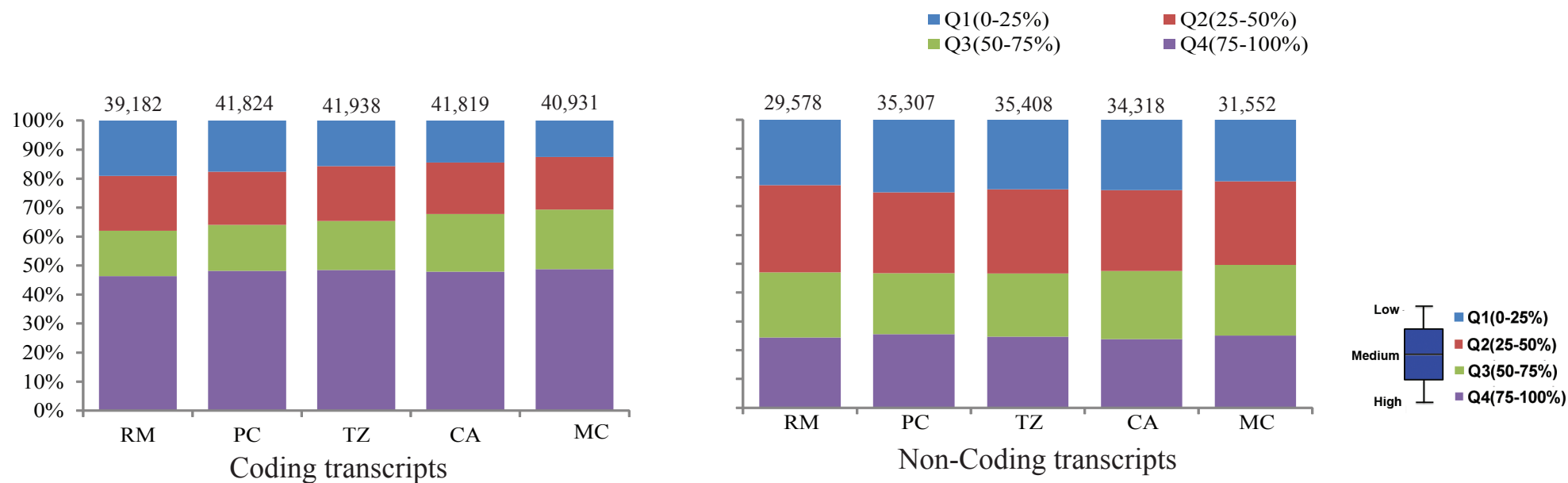

Supplement: Supplementary file 4 — Figure S2. De novo assembly of the reference transcriptome. A) Length distribution of coding and noncoding transcripts across the length bins. Triangles and dots represent the percentages of coding and noncoding transcripts in the length bins, respectively. B) Expression levels displayed differences of 3–4 orders of magnitude. For each tissue layer, transcripts with FPKM ≥0.5 are all replicates. C) In all cases, the coding transcripts (40–50%) are more abundant in the upper ranks of the distribution (Q4) than the non-coding sequences (20–30%). (PDF 468 kb) [file 12864_2019_5560_MOESM4_ESM.pdf]
