# Supplementary material for: Transcriptomic analysis of different tissue layers in antler growth Center in Sika Deer (Cervus nippon)
Source: BMC Genomics. 2019 Mar 5;20:173. doi: 10.1186/s12864-019-5560-1 (PMC6402185; doi:10.1186/s12864-019-5560-1)
Supplement: Supplementary file 5 — Table S3. Summary of 13,203 gene sequences matched with non-redundancy proteins of three closely related species using BLASTX. (DOCX 14 kb) [file 12864_2019_5560_MOESM5_ESM.docx]

**Table S3** Summary of 13,203 gene sequences matched with non-redundancy proteins of three closely related species through BLASTX

| **Species**  **(Genome version)** | **Total proteins** | **Subject** | **Query** | **Subject/ Query** | |
| --- | --- | --- | --- | --- | --- |
| Cattle  (NCBI UMD3.1) | 21,102 | 12,707 (60.2%) | 13,131 (99.5%) | | 96.8% |
| Red deer  (NCBI CerEla1.0) | 19,243 | 9,960 (51.8%) | 12,145 (92.0%) | | 82.0% |
| White tailed deer  (NCBI Ovir.te_1.0) | 21,072 | 12,675 (60.2%) | 13,110 (99.3%) | | 96.7% |
